# Pyroptotic and Necroptotic Cell Death in the Tumor Microenvironment and Their Potential to Stimulate Anti-Tumor Immune Responses

**DOI:** 10.3389/fonc.2021.731598

**Published:** 2021-08-19

**Authors:** Allan Scarpitta, Ulrich T. Hacker, Hildegard Büning, Olivier Boyer, Sahil Adriouch

**Affiliations:** ^1^UNIROUEN, INSERM, U1234, Pathophysiology, Autoimmunity, Neuromuscular Diseases and Regenerative Therapies, Normandie University, Rouen, France; ^2^Department of Oncology, Gastroenterology, Hepatology, Pulmonology, and Infectious Diseases, University Cancer Center Leipzig (UCCL), University of Leipzig Medical Center, Leipzig, Germany; ^3^Institute of Experimental Hematology, Hannover Medical School, Hannover, Germany; ^4^REBIRTH Research Center for Translational Regenerative Medicine, Hannover Medical School, Hannover, Germany; ^5^Department of Immunology and Biotherapy, Rouen University Hospital, Rouen, France

**Keywords:** immunogenic cell death and anti-tumor immunity, pyroptosis, necroptosis, anti-tumor immune responses, tumor immune microenvironment, inflammation and immunity

## Abstract

Cancer remains the second most common cause of death worldwide affecting around 10 million patients every year. Among the therapeutic options, chemotherapeutic drugs are widely used but often associated with side effects. In addition, toxicity against immune cells may hamper anti-tumor immune responses. Some chemotherapeutic drugs, however, preserve immune functions and some can even stimulate anti-tumor immune responses through the induction of immunogenic cell death (ICD) rather than apoptosis. ICD stimulates the immune system by several mechanisms including the release of damage-associated molecular patterns (DAMPs) from dying cells. In this review, we will discuss the consequences of inducing two recently characterized forms of ICD, i.e., pyroptosis and necroptosis, in the tumor microenvironment (TME) and the perspectives they may offer to increase the immunogenicity of the so-called cold tumors and to stimulate effective anti-tumor immune responses.

## Introduction

The term apoptosis was already proposed in 1972 to describe a form “of controlled cell deletion” associated with cytoplasmic condensation and cell fragmentation (1). Later, apoptosis was recognized as the archetypal form of programmed cell death involved in the main physiological processes such as embryonic development, morphogenesis, or cell turnover (2). Apoptosis is now largely described and characterized at the cellular and molecular levels. It involves the activation of initiator caspases *(*e.g., caspase 8, caspase 9), primarily responsible for the activation of downstream effector caspases (e.g., caspase 3) that propagate the death signal by cleaving specific cellular substrates, leading to a controlled dismantling of the cell. Morphological hallmarks associated with apoptosis include cell shrinkage, cell blebbing, formation of apoptotic bodies, and their rapid engulfment by neighboring phagocytic cells. Therefore, apoptosis has been early considered as a regulated and controlled process that avoids inflammation and, therefore, remains immunologically silent. This was opposed to the well-known necrosis, considered instead as a passive, non-programmed form of cell death, resulting in the uncontrolled release of the intracellular content which contains pro-inflammatory molecules that can initiate immune responses. Apoptosis *versus* necrosis have long been regarded as the only conceptual model, even if “unclassical forms” of cell death were reported. It was only recently that the spectrum of cell death mechanism has been deciphered into its broader diversity including notably pyroptosis and necroptosis that constitute the focus of this review. They represent, as for apoptosis, programmed and controlled forms of cell death, but share with necrosis the ability to release pro-inflammatory intracellular molecules that initiate immune responses. Apart from apoptosis, pyroptosis, and necroptosis, other forms of programmed cell death have been described as ferroptosis, parthanatosis, autophagy-dependent cell death, lysosome-dependent cell death, NETosis, or entosis. They will however not be discussed here even if some of them are immunogenic. Also, ICD can also be induced by mechanical or physical treatment and not necessarily from programmed forms of cell death.

First generation chemotherapeutic drugs interfere with cell cycle progression and most of them induce apoptosis in a wide range of cells (3). Consequently, rapidly dividing non-cancer cells, akin to hematopoietic and immune cells, are killed and, due to the induction of apoptosis, anti-tumor immune responses are not induced which represents as what we know now an important risk factor for relapse.

With the advent of the pyroptosis and necroptosis concept, extensive efforts have been made to identify amongst current anti-cancer treatment strategies which include next generation chemotherapeutic drugs, irradiation as well as targeted anti-cancer approaches, those that induce a non-apoptotic cell death. Specifically, the concept emerged that through immunogenic cell death (ICD) accompanied by the release of intracellular adjuvant-like molecules known as damage-associated molecular patterns (DAMPs), a potent anti-tumor adaptive immune response is induced (4).

### Immunogenic Cell Death Is Associated With the Release of Danger Signals That Act as Potent Pro-Inflammatory Immune Adjuvants

In 1994, Polly Matzinger proposed the “danger” model, hypothesizing that the immune system is able to distinguish innocuous circumstances (e.g., commensal bacteria) from threatening situations that are associated with cell death and release of “danger signals” (5). Danger signals encompass, by definition, any molecule invisible to immune cells in normal conditions, which could be liberated or exposed at the cell membrane to alert the immune system in situation of cellular stress, infection, or upon rupture of the cytoplasmic membrane when cells are dying or after a tissue injury. Numerous molecules that are normally present in the intracellular compartment effectively act as danger signals and behave as potent pro-inflammatory adjuvants when released in the extracellular space. A number of molecules have been found to comply to this definition of DAMPs such as, for instance, mitochondrial or nuclear DNA, mitochondrial formylated peptides, heat shock proteins (HSP), F-actin, histones, ATP, or HMGB1 (6).

**Figure 1:**
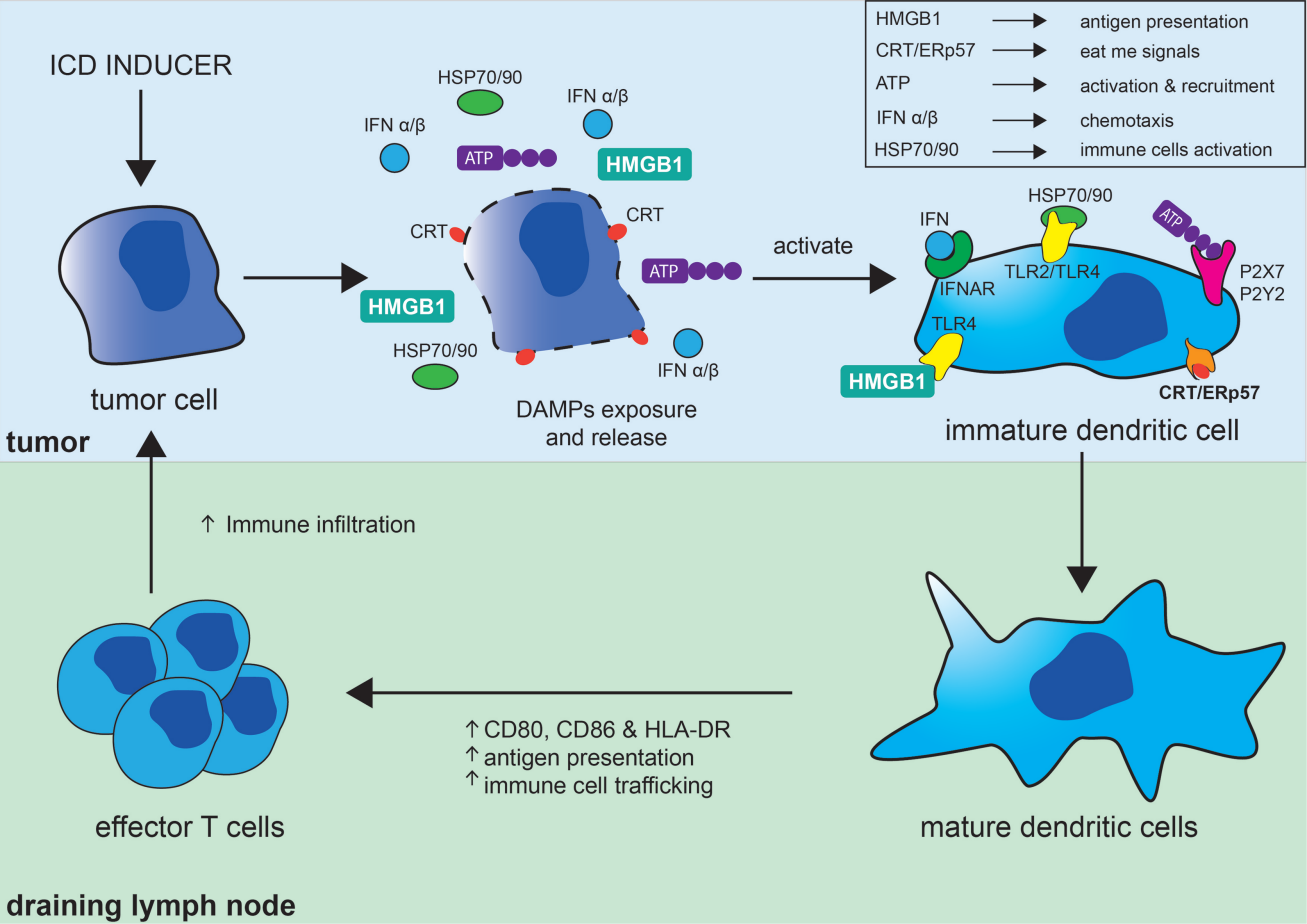
Immunogenic cell death in the tumor context and its consequences on the induction of adaptive immune responses. Immunogenic cell death (ICD) of tumor cell can be induced by different strategies (chemotherapy, oncolytic viruses, radiation therapy, photodynamic therapy, necroptosis, or pyroptosis) and results in the exposure of “eat me signals,” like calreticulin (CRT) and ERp57, and release of DAMPs akin to ATP, HMGB1, HSP70, and HSP90. Interaction of CRT/ERp57 with their receptors acts as “eat me signals” and facilitate engulfment of tumor cells and tumor antigens, interaction of HMGB1 and HSP70/90 with TLRs increase tumor antigens presentation, and fixation of ATP on P2X7 and P2Y2 activate and recruit dendritic cells (DC). ICD and subsequent TLRs stimulation is also accompanied by cytokines release notably by a robust type I interferon (IFNα/β) response, which have broad immune-stimulatory activities. DAMPs and cytokines further participate in the maturation and stimulation of DC leading to increased MHC and co-stimulatory (CD80/CD86) molecules expression, antigen capture and presentation, and their migration to lymph nodes. DC have important implications for the initiation of adaptive immune responses and are involved in the phagocytosis and processing of tumor antigens and their direct and cross-presentation to CD4^+^ and CD8^+^ T cells, respectively, contributing to the activation and proliferation of effector anti-tumor T cells.

Not surprisingly, the release of some of these DAMPs has been used to document the occurrence of ICD and have become the molecular hallmarks associated with ICD. The best characterized DAMPs associated with ICD are CRT/ERp57, HMGB1, HSP 70/90, and extracellular ATP (**Figure 1**). During ICD, the ER-associated molecules CRT and ERp57 re-localize to the outer cytoplasmic membrane and provide an “eat me” signal that promotes phagocytosis by macrophage and dendritic cells. Additionally, exposed CRT represents a specific antigen that can be targeted by cytotoxic T cells. CRT exposure has been suggested to dictate the immunogenicity of dying cancer cells, allowing the capture of tumor antigens by dendritic cells (7). In parallel, the release of the nuclear protein HMGB1 activates dendritic cells notably through its interaction with TLR4 and facilitates the processing and presentation of antigens. Exposition of HSP 70/80 has also immunostimulatory effects mediated by TLR4 and CD14 and can facilitate antigens cross-presentation that is necessary for the presentation of captured tumor-antigens on MHC class I molecules and for the subsequent activation of CD8^+^ cytotoxic immune responses (8). ATP release on the extracellular space is regarded as a potent “find me” signal that is able to exert chemoattractant effects on dendritic cells through the P2Y2 receptor, as well as a potent activator of inflammasome assembly through the stimulation of the P2X7 receptor leading to IL-1β/IL-18 maturation and release (9). The exposition and/or release of these ICD-associated immunostimulatory molecules stimulate adaptive immune responses and possibly potent anti-tumor responses (10). ICD and the subsequent TLR stimulation are also accompanied by the release of cytokines notably by a robust type I interferon (i.e., IFN-α and IFN-β) response. IFNα/β have a wide range of immune-stimulatory activities and participate in the upregulation of major histocompatibility (MHC) molecules on myeloid cells, activation of natural killer (NK) cells, and stimulation and differentiation of effector T cells. Stimulation of dendritic cells has important implications for the initiation of adaptive immune responses and is associated with phagocytosis and processing of tumor antigens, migration towards the draining lymph nodes, upregulation of MHC as well as costimulatory molecules (CD80, CD86), all contributing to an efficient presentation of engulfed tumor antigens to T cells (11) (**Figure 1**).

As mentioned earlier, multiple compounds including classical chemotherapeutic agents, targeted cancer drugs, or irradiation have been recognized to induce ICD under certain conditions. The definition of the molecular hallmarks of ICD has slowly contributed to a shift in the methods used to screen chemotherapeutic drugs for their capacity to induce ICD and to release DAMPs. On the same line, immunocompetent animal models and guidelines to evaluate the immunogenicity of tumor cells exposed to chemotherapy drugs (12) are now replacing animal models based on human tumors engrafted in immunodeficient mice (as recommended by the US National Cancer Institute guidelines), which did not allow to evaluate immunostimulatory effects that some chemotherapeutic drugs may have. This consensual method is based on the evaluation of the ability of drug-exposed killed tumor cells to vaccinate immunocompetent mice and prevent tumor formation elicited by a secondary injection of the same living tumor cell line.

A new field in cancer research has emerged during recent years aiming at a better understanding and eliciting ICD in the tumor context with the aim to favorably manipulate its occurrence, to favor the stimulation of anti-tumor immune responses, and to improve treatment outcomes and overall survival. Given the importance of ICD in other pathophysiological context such as infection as well as in the antigenicity of tumors, studies were performed to characterize ICD morphologically and molecularly.

### Mechanisms Leading to the Induction of Pyroptosis and Necroptosis

**Figure 2:**
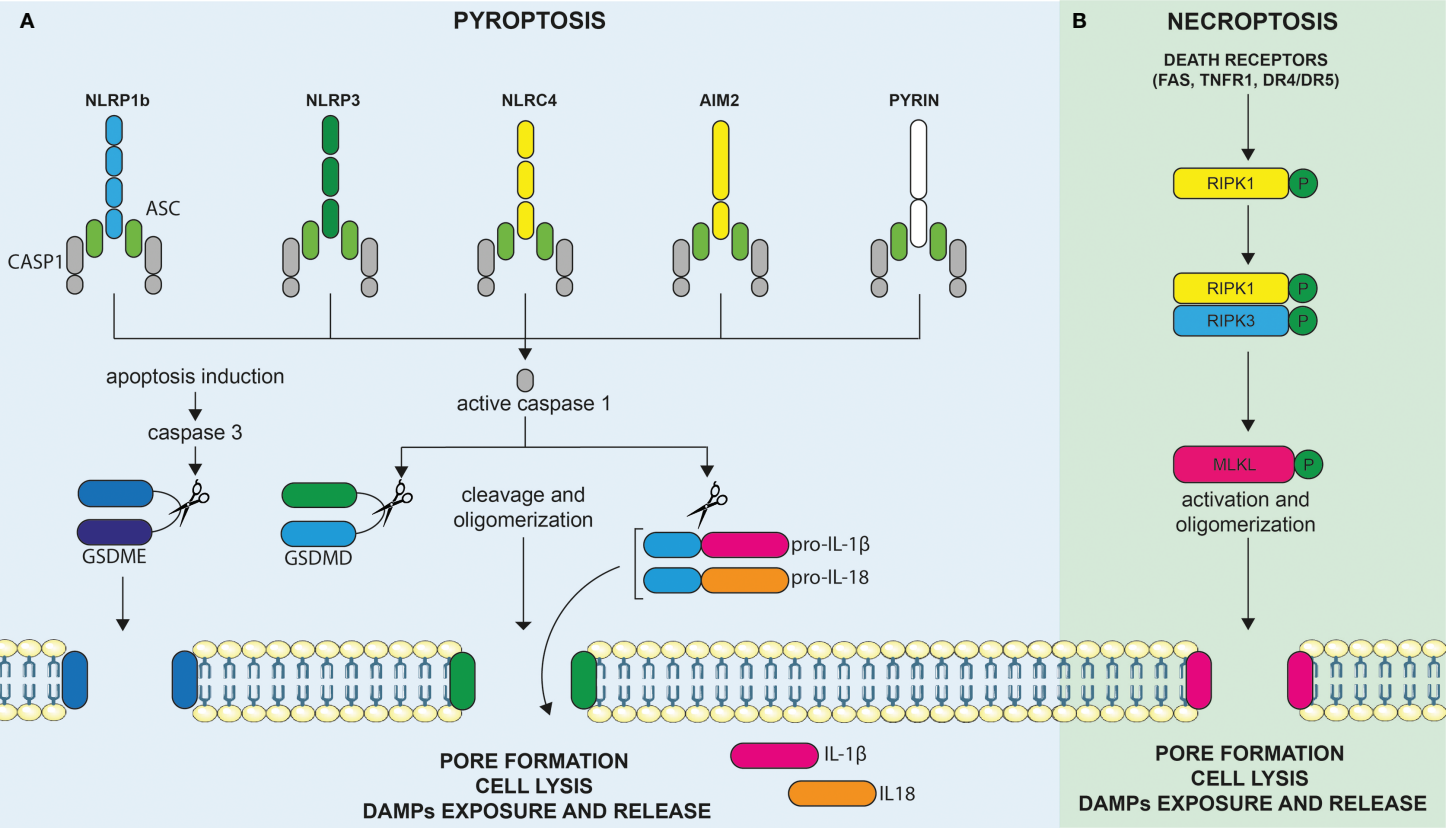
Molecular mechanisms leading to the induction of pyroptosis and necroptosis. **(A)** Cytoplasmic sensors of DAMPs and PAMPs include, but are not restricted to, NOD-like receptors (NLRs), the absent in melanoma-2 (AIM2)-like receptors, and proteins of the pyrin family. Upon binding to their specific DAMPs or PAMPs ligands, these cytoplasmic sensors, such as NLRP1b, NLRP3, NLRC4, AIM2, and pyrin, assemble into an inflammasome multimeric protein complex, containing or not the adaptor proteins ASC, further leading to the recruitment and activation of pro-caspase-1. Activated caspase-1 can cleave and activate the leaderless cytoplasmic cytokines pro-IL-1β and pro-IL-18, leading to their maturation. Active caspase-1 (an others inflammatory caspases) also cleaves gasdermin D (GSDMD), the recently identified pyroptosis executioner, resulting in the liberation of its N-terminal pore forming domain (N-GSDMD) from the auto-inhibitory C-terminal domain. Liberation of the N-GSDMD induces its auto-oligomerization and translocation to the cell membrane to form a non-selective pore that contributes to cell lysis, mature IL-1β/IL-18 release, as well as to the liberation of DAMPs in the extracellular milieu. GSDME, another member of the gasdermin family, is not cleaved by inflammatory caspases but can be cleaved by activated caspase-3 upon induction of apoptosis. Hence, the level of expression of GSDME directly determines the cellular fate between apoptosis or GSDME mediated-pyroptosis. **(B)** Necroptosis represents another inflammatory form of cell death and is mediated by Mixed lineage kinase domain-like (MLKL). Necroptosis can be triggered by the same death receptors (DRs) that are known to induce extrinsic apoptosis (e.g., FAS, TNFR1, DR4/DR5) but occur only when the initiator caspase-8 is blocked, as for instance in tumor or infected cells. In these circumstances, activation of death receptors results in the phosphorylation of RIPK1, RIPK3, and, finally, MLKL. Phosphorylation of MLKL culminates, as for pyroptosis, in the formation of membrane pores (although of smaller size as compared to pyroptosis). Membrane permeabilization to cations further induces cell swelling and osmolysis, ultimately leading to membrane rupture and release of proinflammatory DAMPs in the extracellular milieu, triggering immune stimulation.

In the context of an infection, pyroptosis can be triggered by sensing distinct components of the pathogen, causing inflammasome assembly, caspase-1 activation, and cleavage of Gasdermin family proteins. These activities are eventually leading to pore formations allowing the release of mature IL-1β and IL-18 cytokines (13). Thus, pyroptosis can be defined as an inflammatory cell death involved in host defense against pathogens. It was firstly described in 1992 in infected macrophages (14). The name pyroptosis was, however, given later, after the observation that bacteria-infected macrophages undergo a lytic form of programmed cell death, dependent of caspase-1 activity, and associated with the release of pro-inflammatory IL-1β (15). It appeared, therefore, as a programmed form of cell death, akin to apoptosis, but appeared morphologically more related to necrosis, leading to membrane rupture and to the release of pro-inflammatory molecules. In the infectious context, pyroptosis is triggered by cell-derived DAMPs and/or pathogen-associated molecular patterns (PAMPs) sensed by specific and multiple membrane and cytoplasmic pattern-recognition receptors (PRRs) expressed by innate immune cells. Cytoplasmic sensors of DAMPs and PAMPs include, but are not restricted to, NOD-like receptors (NLRs), the absent in melanoma-2 (AIM2)-like receptors, and proteins of the pyrin family. Upon binding to their specific cell- or pathogen-derived ligands, these cytoplasmic sensors (such as NLRP1b, NLRP3, NLRC4, AIM2, and pyrin) assemble into an inflammasome multimeric protein complex, containing or not the adaptor proteins ASC, further leading to the recruitment and activation of pro-caspase-1 (**Figure 2A**). Mature active caspase-1 plays a central role in the induction of cell death and liberation of pro-inflammatory molecules. Indeed, caspase-1 not only cleaves the cytoplasmic leaderless pro-IL-1β and pro-IL-18 cytokines, leading to their maturation, but also cleaves Gasdermin D (GSDMD), the recently identified pyroptosis executioner (16). Liberation of the N-terminal pore-forming domain of GSDMD (N-GSDMD) induces its auto-oligomerization and translocation to the cell membrane to form a non-selective pore of 10–14 nm inner diameter. This further causes massive ion influx, osmotic cell swelling, and membrane rupture, allowing the release of not only mature IL-1β cytokine (that can directly exit through the pore) but also other pro-inflammatory intracellular contents (17–19). Other mechanisms leading to pyroptosis have also been described. For instance, inflammatory murine caspase-11 was found to directly bind cytoplasmic LPS, leading to its oligomerization and auto-activation (13). In what has been termed as the “non-canonical inflammasome” pathway, activated caspase-11 and, similarly, inflammatory human caspases-4/5 cleave GSDMD directly and induce pyroptosis independently of caspase-1 (16). Apart from GSDMD, other Gasdermin family members display a similar structure, composed of a pore forming N-terminal domain and an auto-inhibitory C-terminal domain. However, the presence of a caspase-1/4/5/11 cleavage site in the long loop linking these domains is unique to GSDMD, indicating that other mechanisms might be involved in the activation of the other family members. Apart from GSDMD, one of the best characterized members of this protein family is GSDME. Interestingly, caspase-3, a well-known executioner of apoptosis, was discovered to cleave and activate GSDME and trigger pyroptosis. As discussed below, this may have important implications for cancer as numerous tumor cells were found to harbor loss-of-function mutations and/or reduced expression of GSDME suggesting a tumor suppressor function. In these cells, the overexpression of GSDME was found to convert apoptosis to pyroptosis and was associated with anti-tumor immune responses (20).

Necroptosis is an inflammatory cell death mediated by mixed lineage kinase domain-like (MLKL) (21). Necroptosis, can be triggered by the same death receptors (DRs) that are known to induce extrinsic apoptosis (e.g., FAS, TNFR1, DR4/DR5) but occurs only when the initiator caspase-8 is blocked, as for instance in tumor or infected cells (22). It is considered as an alternative suicide pathway that triggers caspase-independent death when initiation of apoptosis is inhibited and, therefore, represents an additional back-up mechanism to avoid cell subversion during infection or malignant transformation (18). Downstream of DRs, the molecular pathway, involves the phosphorylation of MLKL by the RIPK1/RIPK3 complex resulting in its oligomerization and its association to the inner leaflet of the plasma membrane (**Figure 2B**). MLKL is the only effector protein known to be able to trigger necroptosis. Phosphorylation of MLKL culminates, as for pyroptosis, eventually in the formation of membrane pores (although of smaller size as compared to pyroptosis). Membrane permeabilization to cations further induces cell swelling and osmolysis (17). Ultimately, this induces the membrane rupture and release of proinflammatory DAMPs into the extracellular milieu, triggering immune stimulation as previously outlined (23).

### Complex Role of Pyroptosis in the TME

GSDMD and GSDME are playing a complex role in TME. Depending on the tumor type and possibly other factors, their expression and function were associated with either tumor regression or tumor progression. As a first example of a possible detrimental role of GSDMD, its expression was found to be upregulated in malignant cells compared to adjacent tissues in non-small-cell lung carcinoma (NSCLC) (24). This was reported for both lung adenocarcinoma (LUAD) and lung squamous cell carcinoma (LUSC) subtypes. Multivariate analyses showed that patients with the highest GSDMD expression in the LUAD group have a lower overall survival, suggesting its potential value as a prognostic factor. This is not the case in patients with LUSC, highlighting the differential role of GSDMD in different tumor contexts. Furthermore, RNA-interference mediated knockdown of GSDMD in tumor NSCLC cell lines attenuates tumor proliferation *in vitro*, as well as *in vivo* in xenografted immunodeficient mice. Moreover, the knockdown of GSDMD favored apoptosis rather than pyroptosis in response to inflammasome activators, displayed lower production of IL-1β, and lower stimulation of the PI3K-Akt pathway (24). Whether GDSMD can regulate the pro-tumorigenic PI3K-Akt pathway in a cell intrinsic manner, directly or indirectly, remains to be explored. These data, however, underscore the detrimental effect of chronic IL-1β liberation in the TME, which is known to recruit myeloid derived suppressor cells (MDSC) and promote neo-angiogenesis. Intriguingly, these tumor cells were indeed found to express not only GSDMD, but also the upstream NLRP3-inflammasome components, akin to myeloid cells, and to display spontaneous signs of inflammasome activation and IL-1β production. Inflammasome activation is known to have detrimental effects in some cancer and associated with a poor prognosis, partly through the unfavorable role of the chronic liberation of IL-1β and IL-18 (25–27). In pancreatic cancer, for instance, NLRP3 signaling has been associated with the recruitment of MDSC and establishment of an immune-suppressive TME (28).

Downstream of inflammasome and GSDMD activation, IL-1β and IL-18 can also have a beneficial effect as they play a pivotal role in the activation of dendritic and natural killer (NK) cells, respectively, and can, thereby, promote anti-tumor immune responses. In line with this notion, the dysregulation of inflammasome signaling and/or consequent reduction of IL-1β and IL-18 production were associated with tumor growth and metastasis in colorectal cancer (28–31). GSDMD, expressed by tumor cells or by cells from the TME, might therefore also be linked to positive effects in some circumstances. In agreement, GSDMD expression in gastric cancer was found to be lower in tumor biopsies compared to adjacent tissues, and its forced lentiviral re-expression in corresponding tumor cells lines was associated with a decrease proliferation *in vitro* as well as *in vivo* following their engraftment in nude mice (32). This suggested that GSDMD expression could limit tumor cell intrinsic proliferation in this type of tumor and sensitize the cells to a pyroptotic cell death. However, the consequence that this may have on the antitumor immune responses obviously could not be evaluated in this immunodeficient mouse model.

As the induction of pyroptosis in tumor cells and cells of the TME hold promise not only to kill tumor cells, but also to induce ICD and anti-tumor responses, a direct induction of pyroptosis using viral vectors encoding the pore forming N-GSDMD represents an interesting perspective. However, the production of viral vectors encoding lytic proteins cause difficulties and generally cell death of the producing cells and a poor viral vector yield. This has, however, been overcome in a first proof-of-principle study by using a specific promoter to control the expression of the toxic gene. This study reported the use of and adeno-associated virus (AAV) vector coding for the pore forming N-GSDMD protein under the control of the P0 Schwann-cell specific promoter. Injection of the corresponding AAV1 vectors in mouse or human Schwannoma tumor implanted, respectively, into immunocompetent and immunodeficient mouse models lead to the significant decrease of tumor growth with no conspicuous signs of toxicity (33). Although immunocompetent mice were used in the syngeneic tumor model, the authors did not evaluate the precise mechanisms leading to the better tumor control and potential immune responses against the tumor.

GSDMD activation is associated with the maturation and liberation of IL-1β with the potentially detrimental effects mentioned earlier. Unlike GSDMD that is cleaved by inflammatory caspases subsequent to inflammasome activation, GSDME is activated by caspase-3. This implies that GSDME-induced pyroptosis is, in principle, not linked to the liberation of IL-1β and may thereby be associated with improved anti-tumor effects. However, a recent study demonstrated that in macrophage cell lines deficient for GSDMD, and that express low levels of GSDME, inflammasome activation can secondarily activate GSDME to form a conduit for IL-1β release without inducing pyroptotic cells death (34). Whether this newly discovered mechanism have any relevance for myeloid cells or tumor cells of the TME remains to be explored.

Caspase-3-dependant apoptosis can be converted to pyroptosis in cells that express sufficient levels of GSDME. This, in essence, holds the potential to convert any pro-apoptotic signal into pyroptosis providing that GSDME is expressed and functional. In agreements, GSDME expression is inactivated in most tumor cells through two complementary mechanisms: downregulation of expression based on epigenic hypermethylation of the promoter, or loss-of-function mutations resulting in an inactive protein unable to form a membrane pore (35–39). Furthermore, methylation of CpG motifs outside the promoter region and within the GDME gene has been described and a growing body of evidence suggests that such methylation patterns might be useful as diagnostic cancer biomarkers. On the other hand, data on GSDME expression levels between cancer and normal samples are controversial: while the majority of translational studies have found downregulated levels of GDME in cancer compared to normal tissue, others have not found such differences. Even more puzzling, there was no clear correlation between GSDME methylation and GSDME expression levels in a number of studies (40). Overall, however, GSDME appears to represent a potent tumor suppressor gene (20). This has been evidenced in primary breast cancer and colorectal cancer using data from the cancer genome atlas database, and GSDME downregulation has been associated with a decrease of overall survival (20). In tumor cell lines, knockout of the corresponding gene in EMT6 and CT26 cells that express GSDME enhances tumor growth. Conversely, lentiviral-mediated expression of full length GSDME decreases the tumor growth of 4T1 and B16 tumor cell lines when grafted into immunocompetent mice. Analysis of immune cell infiltrates underlined the importance of GSDME expression in promoting anti-tumor immune responses characterized notably by tumor-infiltrating NK and CD8^+^ cytotoxic T cells. Interestingly, Granzyme B (GrzB) contained in the cytotoxic granules of NK cells and cytotoxic T cells, and known to represent an important killing mechanism employed by immune cells, was found to directly cleave and activate GSDME independently of caspase-3 activation. Hence, this might be involved in a feedforward mechanism to amplify pyroptosis in the tumor context. In this scenario, a first pro-apoptotic signal induced, for example, by a chemotherapeutic agent would serve as a trigger to initiate pyroptosis in GSDME expressing cells, and secondary recruitment of cytotoxic immune cells would further amplify pyroptosis through the liberation of GrzB and, consequently, the direct activation of GSDME. Some chemotherapeutic drugs are indeed now well described to induce caspase-3 dependent pyroptosis in GSDME expressing tumors (41–43). This may, in part, explain the decreased survival of patients harboring a low GSDME expression in their tumor, which might be more resistant to pyroptotic cell death, and to secondary amplification and protection by the immune responses.

### Necroptosis: Detrimental and Beneficial Consequences in the Tumor Context

In the context of cancer, necroptosis can be considered as a double-edged sword since both positive and negative effects on tumor cell growth and invasion/metastasis have been described in different preclinical models. Components of the necroptosis signaling pathway have been found to be downregulated in some cancers, and this is associated with a poor prognosis (44–47). In agreement, expression of RIPK3, one of the major components of the necrosome complex upstream of MLKL, is repressed by epigenetic mechanisms, and, notably, by methylation of the region surrounding the transcription start site. This was found to be the case in tumor cell lines, in tumor cells from patients with acute myeloid leukemia (AML), as well as in primary breast cancer (47). In the latter type of cancer, RIPK3 expression is reduced in all subtypes analyzed, including in 73% of luminal A group samples, 84% of luminal B, 90% in triple-negative, and in 95% Her2-positives samples. This suggests that the expression of RIP3 is negatively selected during oncogenic transformation, or during tumor growth, and that its down modulation correlates with resistance to chemotherapy. In line with this notion, hypomethylating agents like 5-aza-2′-deoxycytidine (5-AZA) restore RIP3 protein expression in about 70% of the tumor cell lines tested, and could sensitize refractory cell lines to TNFα induced necroptosis as well as to several chemotherapeutic agents in a RIP3-dependent manner (47). Interestingly, this was also studied *in vivo* in a xenograft tumor model where the combination of 5-AZA with doxorubicin was more effective than either treatment alone. However, the impact that this may have on an immunocompetent animal model on the stimulation of the anti-tumor immune response has not been addressed in this humanized model.

Although apoptosis remains the main form of cell death induced by chemotherapeutic agents, accumulating evidences suggest that some drugs, like cisplatin, also induces necroptosis (48). Interestingly, the induction of necroptosis can influence the efficacy and therapeutic effects in some circumstances. Recent works demonstrate that the expression of RIPK3 in esophageal cancer cell lines contribute to cisplatin sensitivity when the apoptotic pathway is deficient or absent (49). In this *in vitro* model, cisplatin treatment induces autocrine production of TNFα, a well-known inducer of the extrinsic apoptotic pathway through the TNFR1. Again, this situation illustrates the notion that blockade of apoptosis triggers necroptosis when the components of the necroptosis pathway are present. Hence, the expression of TNFR1 and of RIPK1/RIPK3/MLKL proteins by tumor cells, in conjunction with the production of TNFα in the tumor microenvironment, could represent novel biomarkers for cisplatin sensitivity in apoptosis-resistant tumors. This may not only be limited to cisplatin as other chemotherapeutic molecules have been described to induce necroptosis by different mechanisms such as shinokin and staurosporin in leukemia or refisibufogenin and 5-FU in colorectal cancer (50, 51).

Regarding the expression of the molecular effectors of necroptosis in tumors, some studies reported a decreased expression of RIPK3, but not RIPK1, in AML (52, 53), while deficiency of both RIPK3/RIPK1 has been reported in colon cancer as compared to non-malignant adjacent tissues (54). In osteosarcoma, overexpression of miR-155-5p has been reported as the mechanism leading to the inhibition of RIPK1 expression and was associated with poor prognosis and increased tumor growth (55). MLKL, the molecular executioner of necroptosis, has also been also found to be downregulated in several types of cancer. In gastric, cervical squamous, and ovarian cancers, a lower expression of MLKL has been correlated with a poor prognosis (44–46). Moreover, MLKL expression has been suggested to represent a potential predictive biomarker in gastric cancer, cervical squamous cancer, and early stage pancreatic adenocarcinoma (45, 46, 56). Taken together, these data suggest that down modulation of RIPK1, RIPK3, or MLKL is associated with poorer prognosis and/or increased tumor aggressiveness, supporting the notion that necroptosis acts as a tumor suppressor and that its repression represents a mechanism for cancer cells to evade cell death.

However, as often in cancer, evidences also exist to suggest the negative consequences associated with the expression of the main actors of necroptosis. For instance, RIPK1 expression in glioblastoma (GBM) has been correlated with a poorer prognosis. The underlying mechanism involves the activation of NF-κB by RIPK1, further leading to the inhibition of P53 activity, thereby, promoting tumor growth (57). In another important study, in the context of pancreatic ductal adenocarcinoma (PDA), contradictory effects of necroptosis were reported when studied *in vitro* and *in vivo*. While, *in vitro* inhibition of the formation of the RIPK1/RIPK3 necrosome complex promoted cancer cell proliferation and aggressiveness, in line with their above-mentioned tumor suppressor role. This was not the case *in vivo* where this was associated instead with tumor rejection (58). These apparently conflicting results were attributed to a negative influence of necroptosis on the composition of the tumor microenvironment. In the mouse model used in this study, based on a genetically inducible tissue specific Kras-dependent oncogenesis, intact RIPK1/RIPK3 signaling favored the recruitment of an immunosuppressive tumor microenvironment (58). Mechanistically, this was dependent on a necroptosis-dependent expression of the CXCL1 chemokine, on the recruitment of myeloid cells, and on their conversion to immunosuppressive and tumorigenic cells by necroptosis-dependent exposition of the nuclear SAP130 protein and consequent activation of the Mincle receptor on myeloid cells. RIPK3 deletion in this PDA model or pharmacological inhibition of RIPK1 induce the recruitment of activated immune cells and favored tumor rejection (58). Hence, in this *in vivo* mouse model, necroptosis seems to promote a chronic, low grade pro-inflammatory response that favors the constitution of immunosuppressive TME. In line with this study, a another report have demonstrated the positive effect *in vivo* of a novel selective small-molecule RIPK1 inhibitor, developed by the GSK company, in a similar mouse model of PDA as well as in organoid human models (59). In this study, RIPK1 was demonstrated to be upregulated in immunosuppressive tumor-associated macrophages (TAM). Its inhibition reprogrammed TAM towards an immunogenic phenotype, induced T cell activation and T helper differentiation into anti-tumoral Th1/Th17 phenotypes, and improved anti-tumor immunity.

These studies highlight the importance of studying necroptosis consequences not only in the tumor cells themselves but also on the cells of the TME and on the induction of anti-immune responses. As evoked earlier, necroptosis leads to the release of DAMPs and stimulation of immune cells by different molecular pathways. However, recent studies show that the release of DAMPs upon necroptosis induction is not sufficient alone to induce efficient cross-priming of CD8^+^ T cells and robust adaptive immunity (60). Instead, this seems to require complex and coordinated molecular signaling pathways that involve the RIPK1-dependant activation of NF-κB and transcriptional activity, despite the rapid entry into cell death, that results in the parallel expression of pro-inflammatory genes and cytokines (57, 58). Interestingly, the direct intratumoral induction of necroptosis using an AAV vector encoding for a constitutively active form of RIPK3 induce necroptosis in a part of the tumor cells, improves tumor immunogenicity, and synergizes with immune checkpoint blockade to promote a durable tumor clearance (61). In a similar strategy, the direct intratumoral delivery of mRNA coding for the necroptosis executioner MLKL, followed by *in vivo* electroporation at the injection site, reduced tumor growth and metastasis, synergized with immune checkpoint blockade, and improved antitumor immunity in murine melanoma and colon carcinoma models (62). In this study, MLKL mRNA electroporation *in vivo* was demonstrated to induce CD4^+^ and CD8^+^ T cell responses directed against tumor antigens that were responsible for tumor control. Taken together, these data in mouse models indicate that fostering necroptosis directly in tumor cells may represent an attractive novel strategy to promote tumor antigens release, their efficient presentation, and the induction of immune responses against tumor cells. Whether favoring the induction of necroptosis rather than apoptosis is associated with the induction of anti-tumor immune responses and/or better treatment outcomes in patients with established tumors remain to be fully explored.

## Perspectives

Pyroptosis and necroptosis play complex and multifaceted roles, depending on the cancer type and tumor context. Yet, the majority of the mutations in the gene coding for GSDME found in tumor tissues corresponds to the loss of functions mutations, suggesting a tumor suppressor role (20). The main proteins involved in the induction of necroptosis are also found to be dysregulated in different human cancers suggesting that necroptosis represent another important factor that contributes to tumor clearance and/or to the restriction of their malignancy. As pyroptosis and necroptosis are both involved in the induction of ICD within the TME, it is tempting to speculate that they contribute to the induction of anti-tumor immune responses. The strongest evidences that the immune system can be beneficial to treat or even cure cancers come from the success of immune checkpoint blockade using monoclonal antibodies directed against CTLA-4 or PD-1/PD-L1 (63, 64). Importantly, the treatment approach is most efficient in cancers that are highly immunogenic (i.e., melanoma, renal cell carcinoma, and others). Combinatorial approaches to increase the efficacy of immune checkpoint blockade in less immunogenic and immunologically “cold” tumors are currently being tested in both preclinical and clinical trials. Given the long-term beneficial effect of the induction of anti-tumor immune responses and their potential amplification with anti-immune checkpoints, several therapeutic strategies aim to stimulate ICD rather than the tolerogenic apoptotic forms of cell death. This comprises the selection of chemotherapeutic molecules that induce ICD rather that apoptosis, the evaluation of nanoparticles based-treatments, advanced physical techniques, as well as the development of oncolytic viruses (11). Manipulation of pyroptosis or necroptosis in cancer, in conjunction with the preservation of immune functions, could represent a novel promising option to induce tumor cell death, acute inflammation, tumor antigens release, their capture and presentation by mature dendritic cells, and eventually to trigger an efficient stimulation of adaptive anti-tumor T cells. This has, at least, been proven in proof-of-concept studies in animal models, using, for instance, AAV vectors coding for the constitutively active form of RIPK3. This study elegantly demonstrates that the intratumoral induction of necroptosis using this strategy promotes effective immune responses and durable tumor clearance in combination with immune checkpoints blockade (61). Similarly, induction of pyroptosis using AAV vectors coding for the pore forming N-GSDMD protein under the control of a tumor specific promoter leads to the significant decrease of tumor growth in a schwannoma model (33). Additionally, induction of pyroptosis or necroptosis of cells that constitute the tumor microenvironment might potentially be used to rewire immunosuppressive TME and reduce tumor resistance to established therapies. However, some recent works also highlighted the potential negative consequences of pyroptosis/necroptosis in the tumor context stressing the need to further study the consequences of this strategy when used alone or in combination with other cancer treatments.

## Author Contributions

AS and SA: writing – original draft. AS, UH, HB, OB, and SA: writing – review and editing. UH, HB, OB, and SA: funding acquisition. All authors contributed to the article and approved the submitted version.

## Funding

This work was supported by grants from the Agence Nationale de la Recherche (ANR) to OB and SA (ANR-20-CE92-0011) and the Deutsche Forschungsgemeinschaft (DFG) to UTK and HB (BU1310/9-1).

## Conflict of Interest

The authors declare that the research was conducted in the absence of any commercial or financial relationships that could be construed as a potential conflict of interest.

## Publisher’s Note

All claims expressed in this article are solely those of the authors and do not necessarily represent those of their affiliated organizations, or those of the publisher, the editors and the reviewers. Any product that may be evaluated in this article, or claim that may be made by its manufacturer, is not guaranteed or endorsed by the publisher.

## Abbreviations

DAMPs, damage-associated molecular patterns; GSDMD, gasdermin D; GSDME, gasdermin E; ICD, immunogenic cell death; MLKL, mixed lineage kinase domain-like; PAMPs, pathogen-associated molecular patterns; PRRs, pattern-recognition receptors; TME, tumor microenvironment
